# Supplementary material for: Hippocampus supports multi-task reinforcement learning under partial observability
Source: Nat Commun. 2025 Oct 30;16:9619. doi: 10.1038/s41467-025-64591-9 (PMC12575782; doi:10.1038/s41467-025-64591-9)
Supplement: Supplementary file 1 — Supplementary Information [file 41467_2025_64591_MOESM1_ESM.pdf]

## Supplementary Information

| | Experimental task | RL agent |
|---|---|---|
| Credit assignment | Reward-based learning[1] | Q-learning + BPTT (within trial) |
| Main tasks | Allo, Ego | Allo, Ego |
| Sub-tasks | North, South | North, South |
| Task inference | Trial-and-error | HC + output layers[2] |
| Action selection | Striatum[1] | Separate ego and allo action outputs |
| Policy | Unclear[1] | $\epsilon$-greedy |
| Sensory input | External cues and maze | Simplified cues and 2D maze |
| Training regime | Block-wise | Block-wise |
| Trials per block | 25-50 | 25 |
| Total number of trials | 800 | 15000 |
| Trained params. | Synapses[1] | Weights and biases |
| Env. observability | Inherently limited[1] | Partial (3x3) or full (9x9) view |
| Sensory repres. | Latent from complex input | Latent from (simplified) 2D world view |
| Architecture | Full brain | Focus on hippocampus |
| Neural activity | CA1 tetrode recordings (sub-sampling) | Full access to CA1 activity |

**Supplementary Table S1.** Mapping between experimental setting and our RL modelling framework. [1] These elements remain open to interpretation, and we offer our own perspective on how the brain may achieve these components. [2] Although our RL agents still require trial-and-error to infer the task, this inference process is made easier by assuming separate ego and allocentric output action heads.

| Name | Value |
|---|---|
| Discount factor, $\gamma$ | 0.9 |
| Adam learning rate, $\alpha$ | 0.001 |
| Epsilon-greedy, $\epsilon$, max-min | 0.3 - 0.05 |
| Batch size | 1 |
| Neural layers | 3 HC + 1 output |
| Input size | 9/81 |
| Hidden size | 50 |
| hcDRQN hidden size | 50 |
| Output size | 3 |
| Target update counter | 25 |

**Supplementary Table S2.** Hyperparameters used to run the experiments given in the paper.

| Model | Spatial information $\uparrow$ (bits) | Sparsity index $\downarrow$ | Spatial coherence $\uparrow$ |
|---|---|---|---|
| hcDQN | $1.38 \pm 0.10$ | $0.406 \pm 0.017$ | $0.656 \pm 0.014$ |
| hcDQN (full) | $1.27 \pm 0.08$ | $0.445 \pm 0.021$ | $0.637 \pm 0.019$ |
| **hcDRQN** | $\mathbf{1.88} \pm 0.16$ | $\mathbf{0.308} \pm 0.045$ | $\mathbf{0.670} \pm 0.024$ |
| hcDRQN (full) | $0.83 \pm 0.09$ | $0.599 \pm 0.030$ | $0.558 \pm 0.021$ |

**Supplementary Table S3.** Spatial metrics for each model (mean $\pm$ SEM).

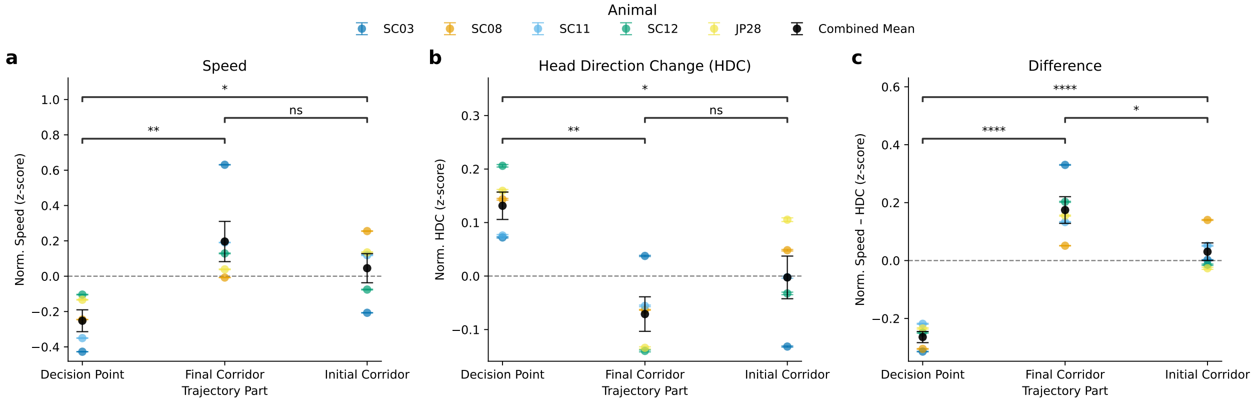


**Supplementary Figure S1. Speed and head direction change in animals.** This figure compares the animals' speed and head direction change in three zones of the maze: the initial corridor, the decision point, and the final corridor. **a**, Normalised speed. The results show that the animals move at a lower speed when at the decision point compared to the corridor zones, indicating a slowdown as they make navigational choices. **b**, Normalised head direction change. It is significantly higher at the decision point than in the corridors, suggesting that the animals engage in more head movements, which is likely a reflection of turning towards the final arm. In the corridor zones, the animals tend not to look around extensively, supporting the idea that they do not constantly scan the environment during forward navigation, thus suggesting a degree of partial observability. **c**, Difference between speed and head direction change (HDC). It highlights when speed/HDC are different. Independent two-sample, two-sided t-tests, data are presented as mean values $\pm$ SEM ($n = 5$). *: $p < 0.05$, **: $p < 0.01$, ***: $p < 0.001$, ****: $p < 0.0001$, ns indicates no significant. Source data are provided as a Source Data file.

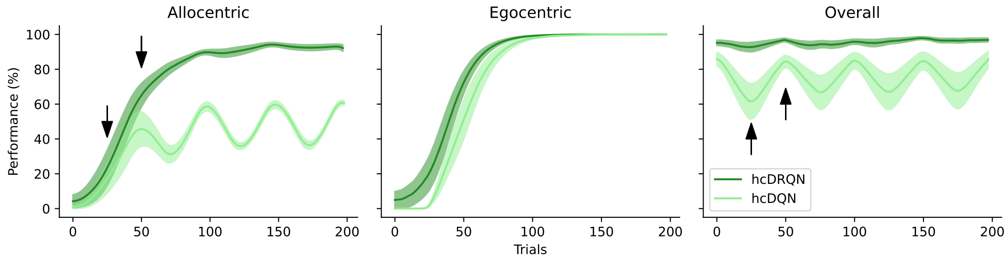


**Supplementary Figure S2. Performance of hcDRQN and hcDQN using a single head output and task ID.** Although both models receive task ID information, only hcDRQN successfully solves both allocentric and egocentric tasks. The arrow indicates the switching point between north and south starting locations. Data are presented as mean values $\pm$ SEM ($n = 5$). Source data are provided as a Source Data file.

| Condition | Comparison | $p$ |
|---|---|---|
| Allo N | hcDRQN vs. hcDQN | $4.09 \times 10^{-16}$ (****) |
| Allo S | hcDRQN vs. hcDQN | $9.74 \times 10^{-13}$ (****) |
| Ego N | hcDRQN vs. hcDQN | $3.44 \times 10^{-5}$ (***) |
| Ego S | hcDRQN vs. hcDQN | $1.37 \times 10^{-7}$ (****) |
| Overall | hcDRQN vs. hcDQN | $7.29 \times 10^{-26}$ (****) |
| Overall | hcDRQN vs. hcDRQN (full) | $9.97 \times 10^{-1}$ (ns) |
| Overall | hcDRQN vs. hcDQN (full) | $1.39 \times 10^{-32}$ (****) |

**Supplementary Table S4.** Independent two-sample $t$-tests for Allo/Ego panels and Overall (bars with per-seed overlays) shown in Fig. 4c. Significance: ns ($p > 0.05$), * ($p \leq 0.05$), ** ($p \leq 0.01$), *** ($p \leq 0.001$), **** ($p \leq 0.0001$).

| Condition | Comparison | $p$ |
|---|---|---|
| Allo N vs Allo S (allo) | hcDRQN vs. hcDQN | $3.93 \times 10^{-119}$ (****) |
| Ego N vs Ego S (ego) | hcDRQN vs. hcDQN | $1.65 \times 10^{-54}$ (****) |
| Allo N vs Ego N (a1e1) | hcDRQN vs. hcDQN | $5.95 \times 10^{-111}$ (****) |
| Allo N vs Ego S (a1e2) | hcDRQN vs. hcDQN | $1.62 \times 10^{-103}$ (****) |
| Allo S vs Ego N (a2e1) | hcDRQN vs. hcDQN | $9.27 \times 10^{-112}$ (****) |
| Allo S vs Ego S (a2e2) | hcDRQN vs. hcDQN | $1.96 \times 10^{-111}$ (****) |
| Overall | hcDRQN vs. hcDQN | $1.14 \times 10^{-61}$ (****) |
| Overall | hcDRQN vs. hcDRQN (full) | $7.98 \times 10^{-2}$ (ns) |
| Overall | hcDRQN vs. hcDQN (full) | $1.91 \times 10^{-54}$ (****) |

**Supplementary Table S5.** Independent two-sample $t$-tests for heatmap pairwise error panels and Overall shown in Fig. 4d. Significance: ns ($p > 0.05$), * ($p \leq 0.05$), ** ($p \leq 0.01$), *** ($p \leq 0.001$), **** ($p \leq 0.0001$).

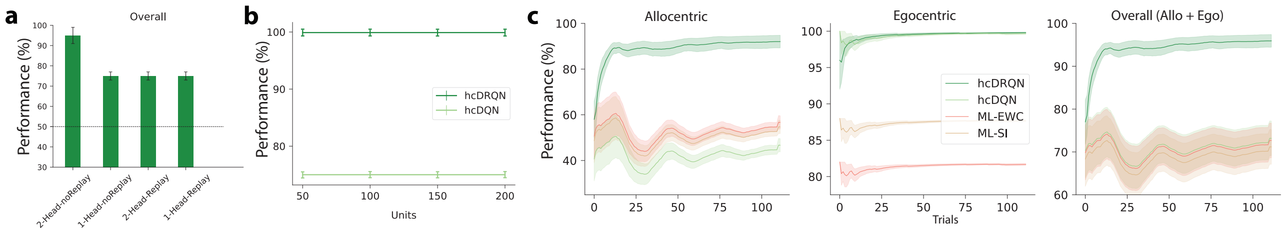


**Supplementary Figure S3. Model performance with/without multi-head, replay buffer, different numbers of CA3 neurons and model learning curves. a**, Only hcDRQN model trained with two heads and without experience replay is able to solve all the tasks while all the variants with one/two head and with/without experience replay reach only 75% performance. Alternatively its possible to train agents with only one head but providing them with task-specific information Fig. S2. **b**, Changing the number of CA3 neurons has no effect on the final performance. **c**, Learning curves for hcDQN, hcDRQN, ML-EWC, ML-SI. Data are presented as mean values $\pm$ SEM ($n = 5$). Source data are provided as a Source Data file.

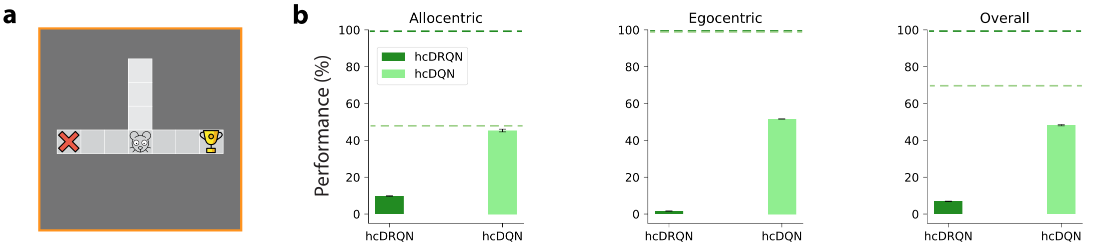


**Supplementary Figure S4. Fully observable models require the cues all the time. a**, Minigrid environment showing full view size with cue removal with agent at the decision point. **b**, Task performance with cue removal after the agent reaches the decision point for both hcDQN and hcDRQN trained with full observability. Dotted line represents performance of partial view models. Data are presented as mean values $\pm$ SEM ($n = 5$). Source data are provided as a Source Data file. Icons used in panel a are released as open-source by OpenMoji.

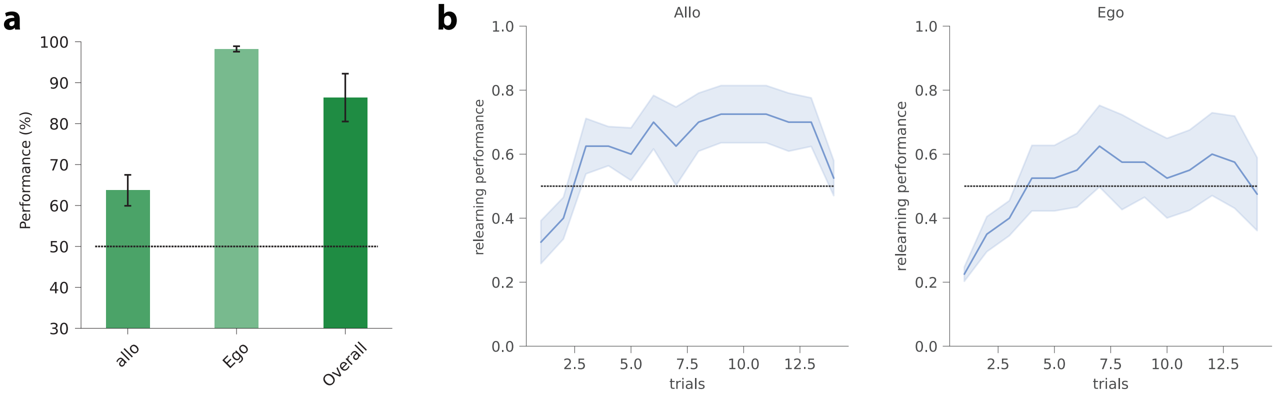

**Supplementary Figure S5. hcDRQN model performance mid learning and animal learning curves.** **a**, Allocentric, egocentric and overall performance for hcDRQN model taken during middle phase of training. **b**, Learning curve for animal data averaged across allo/egocentric blocks. They resemble the ones from the hcDRQN model. Independent two-sample, two-sided t-tests, data are presented as mean values $\pm$ SEM ($n = 5$). *: $p < 0.05$, **: $p < 0.01$, ***: $p < 0.001$, ****: $p < 0.0001$, ns indicates no significant. Source data are provided as a Source Data file.

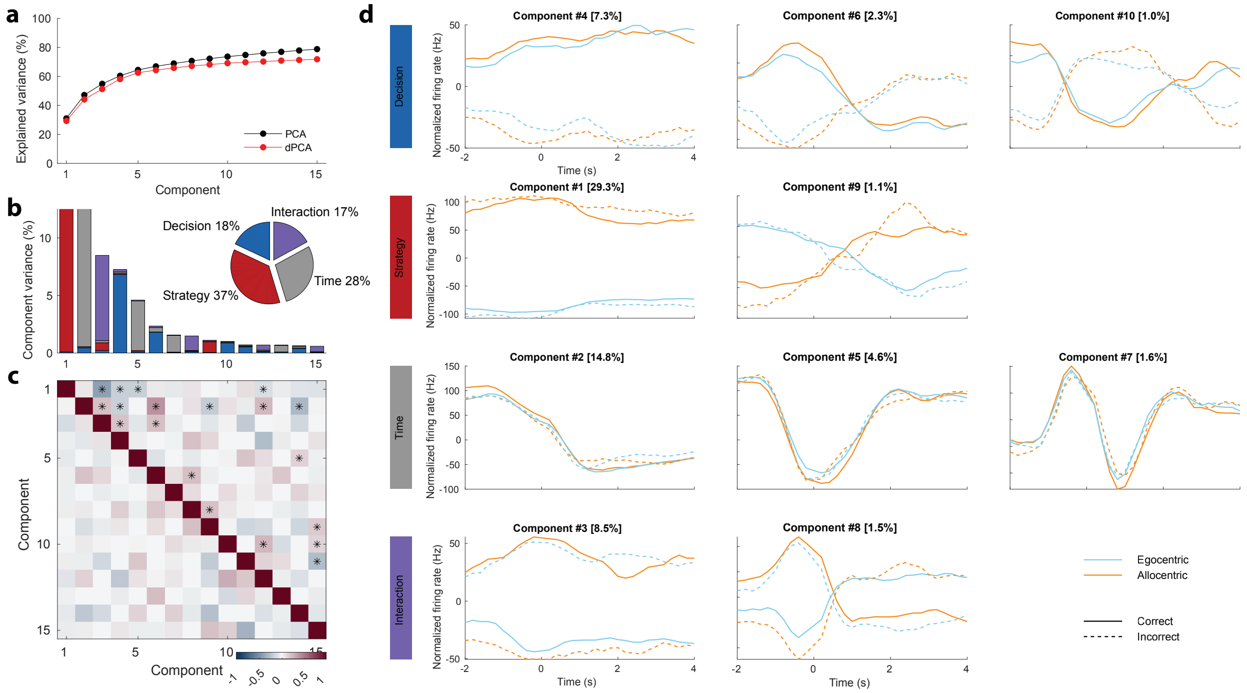

**Supplementary Figure S6. Demixed PCA with all the components for animal data.** **a**, Cumulative explained variance of PCA (black) against dPCA (red). **b**, Variances explained by each demixed principal component. In the pie chart, the total data variance is divided per task-specific variable. **c**, Dot products between all pairs of demixed principal components is shown in the upper-right triangle. Stars denote the pairs that are significantly non-orthogonal. Correlation among all demixed principal component pairs is displayed in the lower-left triangle. **d**, Top row: first three decision components; second row: first two strategy components; third row: first three time components; last row: first two decision/strategy interaction components. Figure produced using code made available by[?] (follows a similar structure to the figures available in the original dPCA paper).

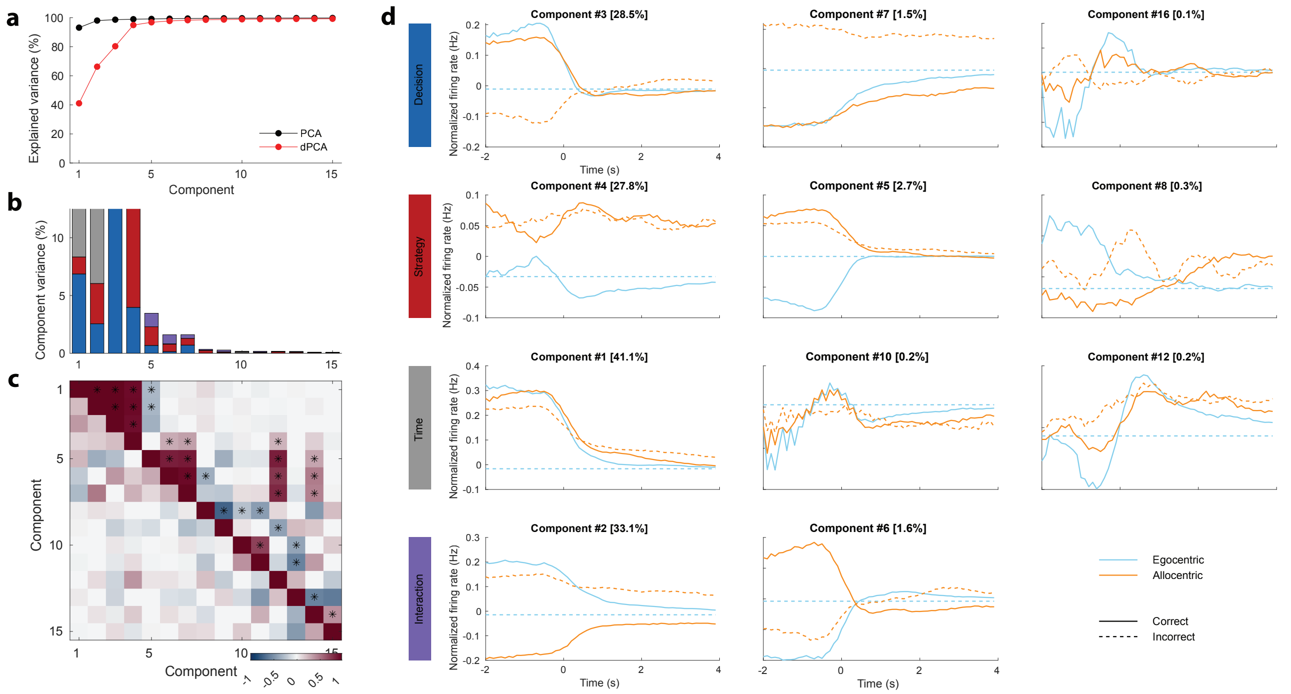

**Supplementary Figure S7. Example of typical demixed PCA with all components for hcDRQN. a,b,c,d**, As in the previous figure. This is shown for one seed only due to computational reasons, but the results are consistent across seeds.

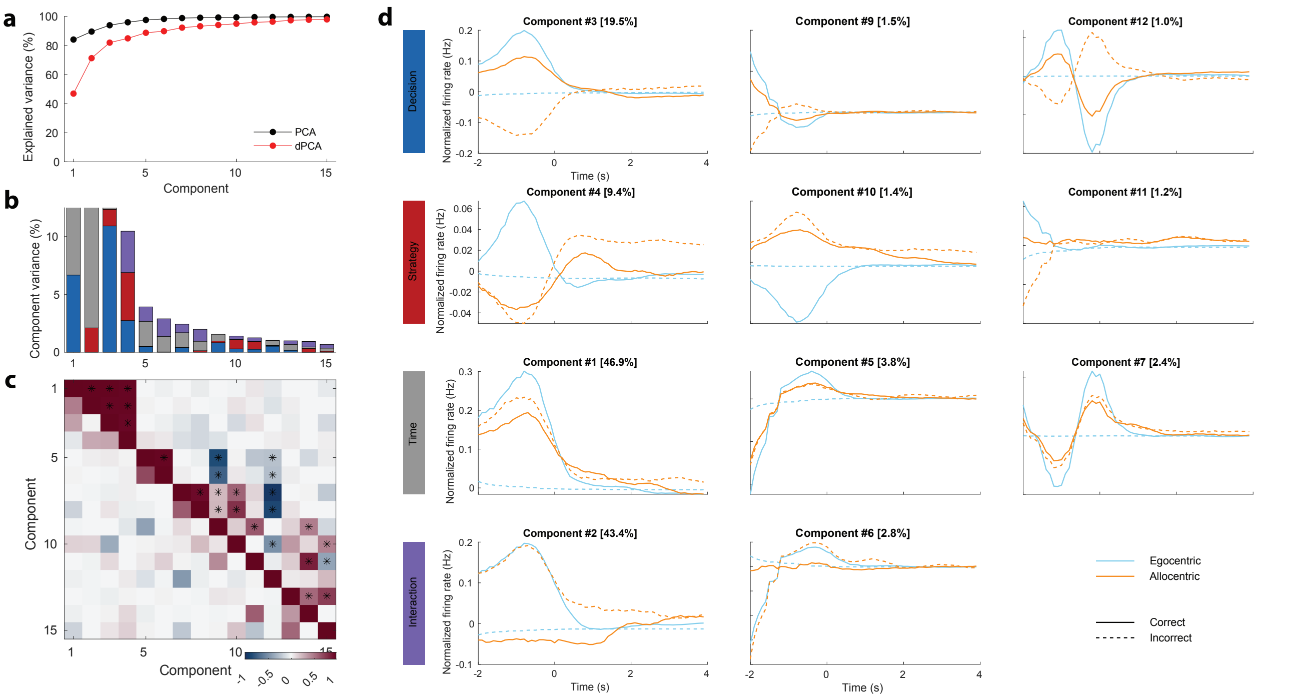

**Supplementary Figure S8. Example of typical demixed PCA with all components for hcDQN. a,b,c,d**, As in the previous figure. This is shown for one seed only due to computational reasons, but the results are consistent across seeds.

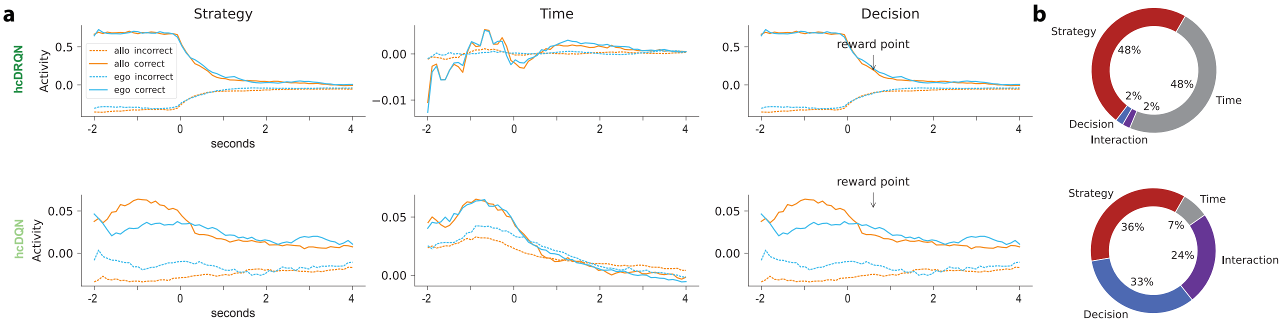


**Supplementary Figure S9. Outcome, strategy and temporal neural dynamics in RL agents with full view.** **a**, Demixed PCA components corresponding to Decision - Correct vs Incorrect, Strategies - Allocentric and Egocentric. Qualitatively, full view hcDRQN shows similar trends for decision and strategies, but fails to capture the time component. Full view hcDQN presents mixing activity for strategy components. **b**, Percentage of explained variance for different components.

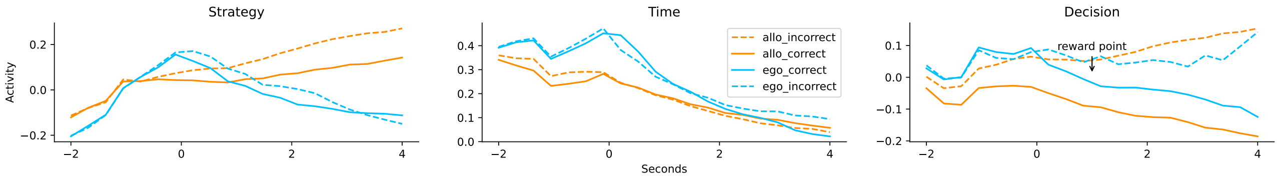


**Supplementary Figure S10. Demixed PCA (dPCA) of the hcDRQN model in a maze environment without cues.** Compared to the version with cues (Fig. 3a, first row), the separation of the components for strategy and decision is less clear, suggesting that cues play a critical role in enhancing the model's ability to differentiate between these task-related variables.

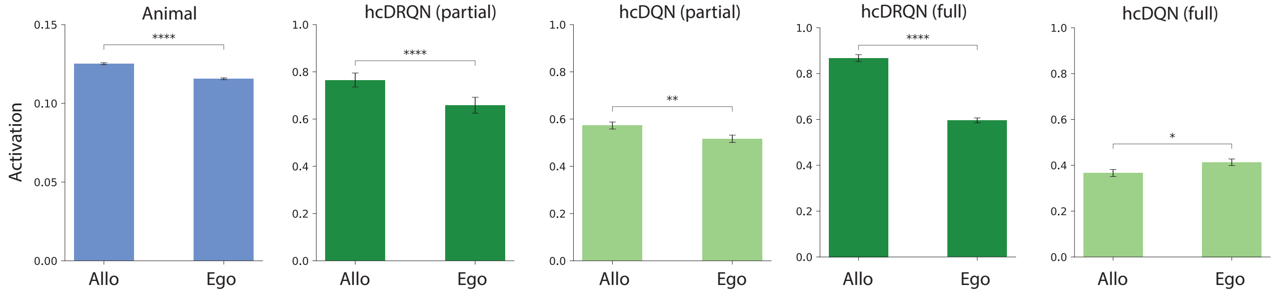


**Supplementary Figure S11. Task-specific neural activity.** Comparing neural activity between allocentric vs egocentric tasks shows that in both animal and partial view models, allocentric activity is higher. In the full view models, hcDRQN follows the previous pattern while hcDQN shows higher activity in egocentric tasks. Data are presented as mean values $\pm$ SEM ($n = 5$). Source data are provided as a Source Data file.

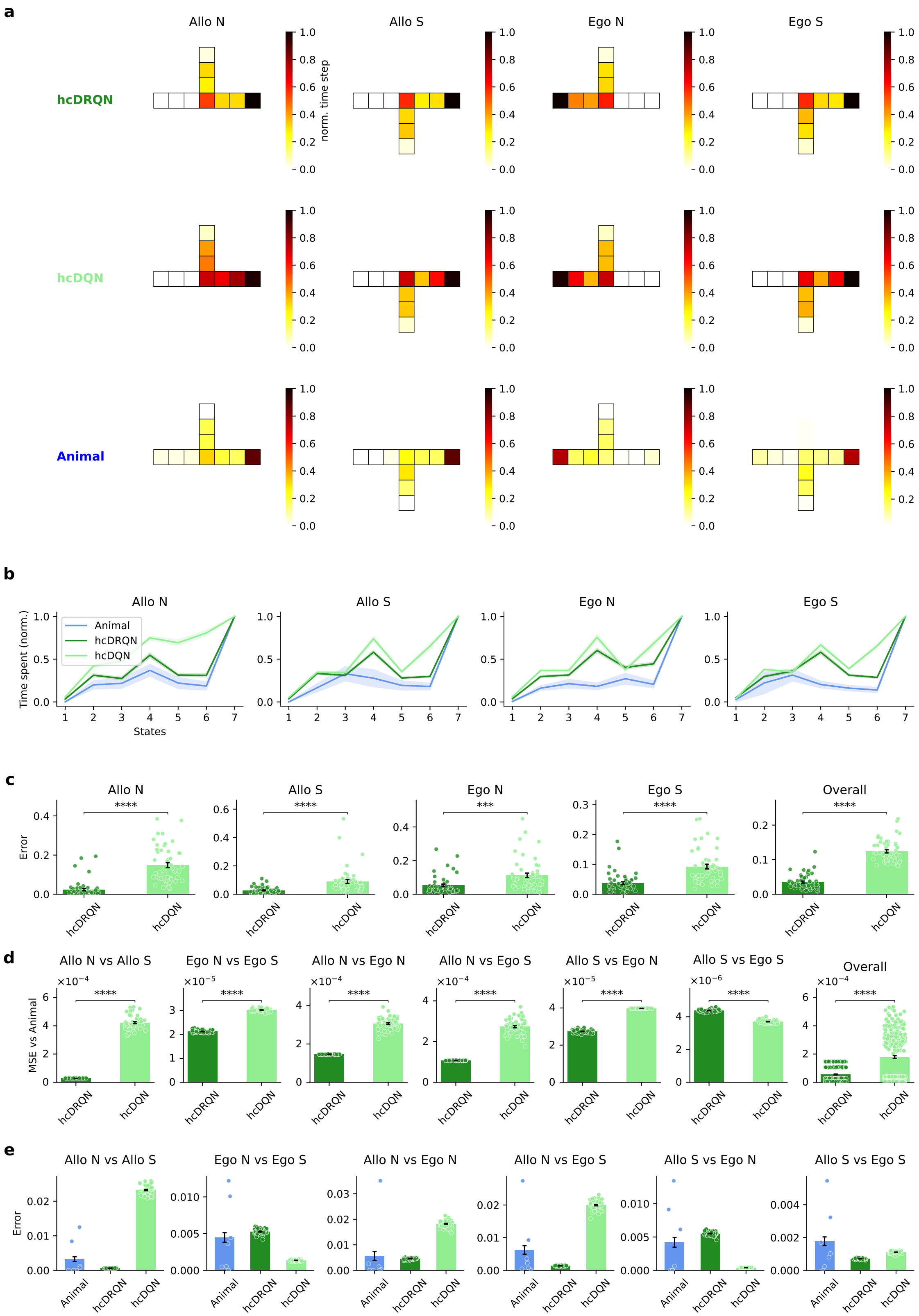


**Supplementary Figure S12. Trajectory maps for RL models trained with full observability.** **a**, We repeat the same analysis as done in Fig. 4 with full view models. **b**, Time spent on each state normalised to the time spent on the final (terminal) state in models and animals. **c**, Error between agents and animals for each strategy shows that hcDRQN better captures animal behaviour. **d**, Error between models and animals across all possible task-pairs shows mixed behaviour in terms of which model provides a closer match to animal behaviour. **e**, Error between all possible task-pairs shows that full view hcDRQN errors are lower for Allo North task ratios while in full view hcDQN are lower for Allo South task. Data are presented as mean values ± SEM ($n = 50$). ****: $p < 0.0001$, ns indicates no significant (independent two-sample, two-sided t-tests across models). Source data are provided as a Source Data file.

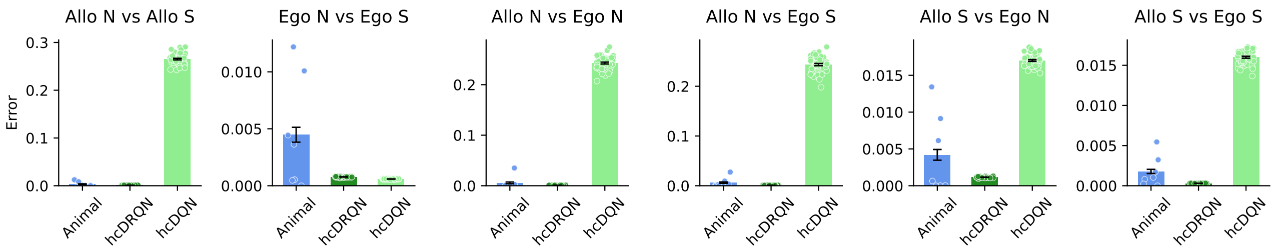


**Supplementary Figure S13. Error between animal and model trajectory-occupancy maps (cf. Fig. 4).** Error was calculated between all possible task-pairs for both animal and models (1 refers to North and 2 refers to South starting position). Data are presented as mean values ± SEM ($n = 50$). Source data are provided as a Source Data file.

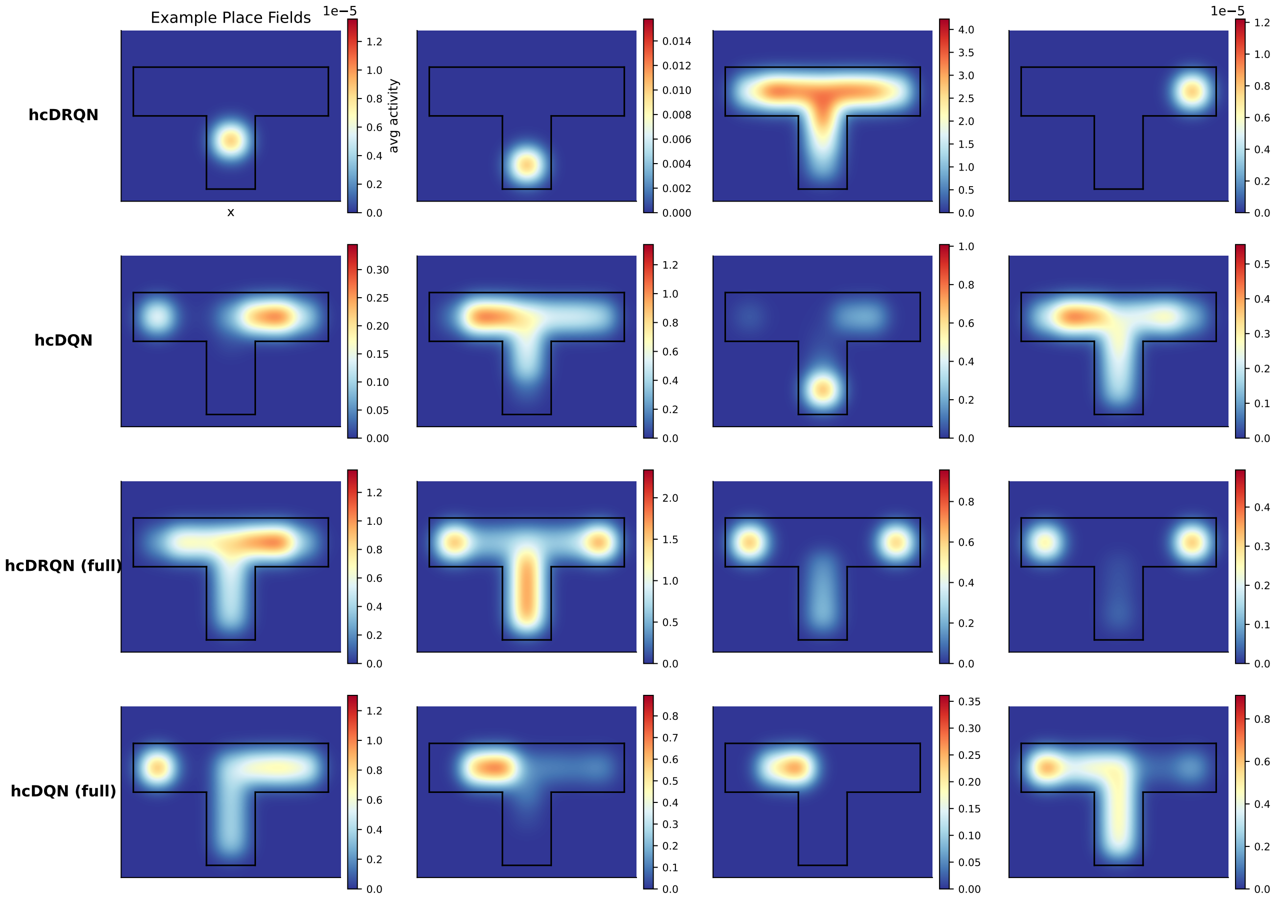


**Supplementary Figure S14. Example place fields across models.** Each row shows four example place fields from each model type: hcDRQN, hcDQN, and their full-view variants (hcDRQN (full) and hcDQN (full)). Each column corresponds to a single neuron. Place fields are overlaid on the T-maze geometry, highlighting how different models develop spatially tuned representations.

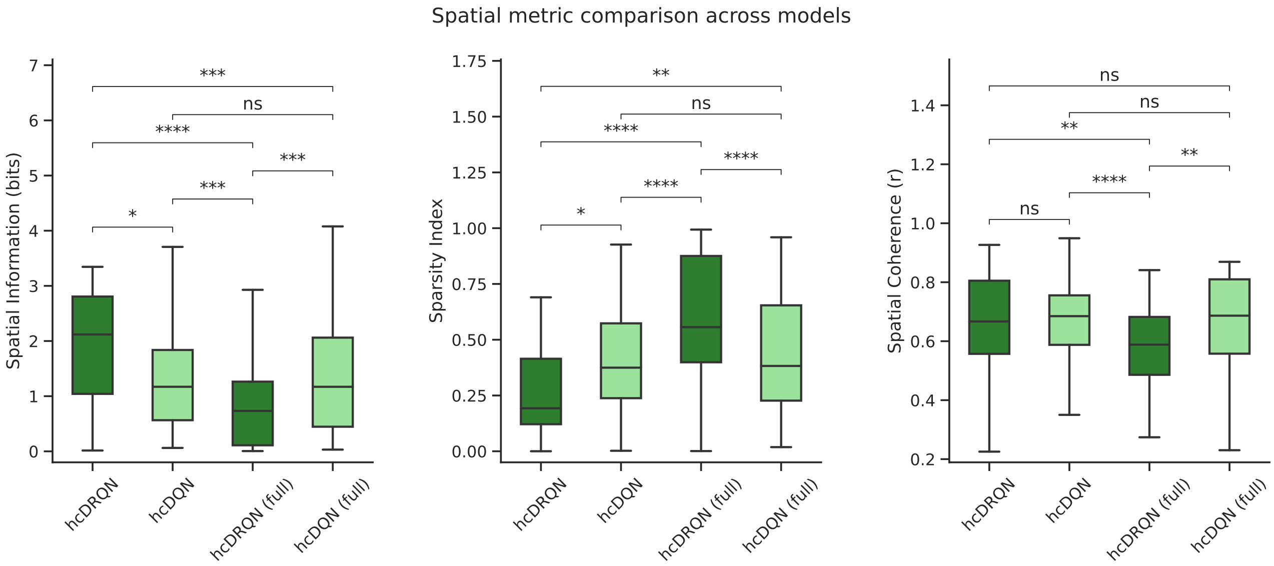


**Supplementary Figure S15. Comparison of spatial coding metrics across model types.** Box plots show distributions of three standard spatial tuning metrics computed across all CA1 neurons for each model: spatial information (left), sparsity index (middle) and spatial coherence (right). The hcDRQN model shows significantly higher spatial information and lower sparsity index compared to the other variants, indicating more spatially selective and compact representations. Coherence is also slightly higher (ns) in hcDRQN, reflecting smoother spatial tuning. These results suggest that hcDRQN captures spatial structure more effectively than both its feedforward counterpart (hcDQN) and full-view versions. Box plots show median (center line), interquartile range (box = Q1–Q3), and whiskers extending to the most extreme data within 1.5×IQR from the hinges; points beyond the whiskers are considered outliers and are not displayed. Thus the plotted min/max are the whisker endpoints, not the global extrema. Two-sided independent-samples t-tests on per-neuron SI values ($n = 200$). *: $p < 0.05$, **: $p < 0.01$, ***: $p < 0.001$, ****: $p < 0.0001$, ns indicates no significant. Source data are provided as a Source Data file.

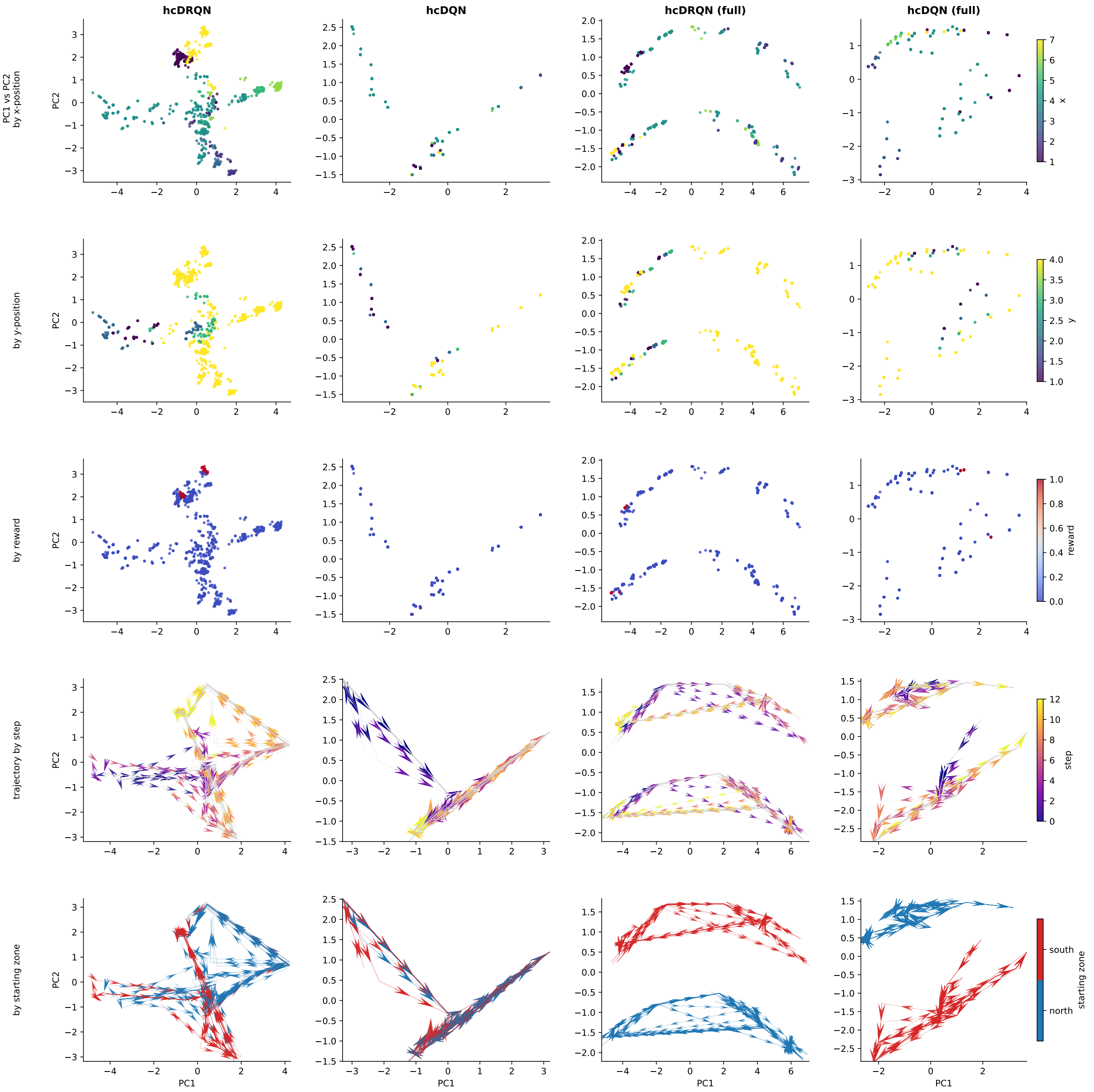


**Supplementary Figure S16. PCA-based visualisation of spatial features and trajectory structure across model types.** Each column shows results from different models (hcDRQN, hcDQN, hcDRQN (full), hcDQN (full)). The top three rows project neural activity onto the first two principal components (PC1 and PC2), colour-coded by x-position (row 1), y-position (row 2) and reward magnitude (row 3). These projections highlight how spatial variables are encoded in the hidden activity of the CA1 layer. The hcDRQN model shows stronger spatial representation by position and reward compared to hcDQN. Full-view models (right columns) exhibit two clear clusters in PC space. The bottom two rows show trial trajectories in PC space, colour-coded by step number (row 4) and starting zone (north vs south; row 5). In full-view models, the two PC clusters align with starting zones, suggesting that the full-view input strongly shapes neural dynamics. In contrast, hcDRQN trajectories show a more continuous and compact representation of spatial structure.

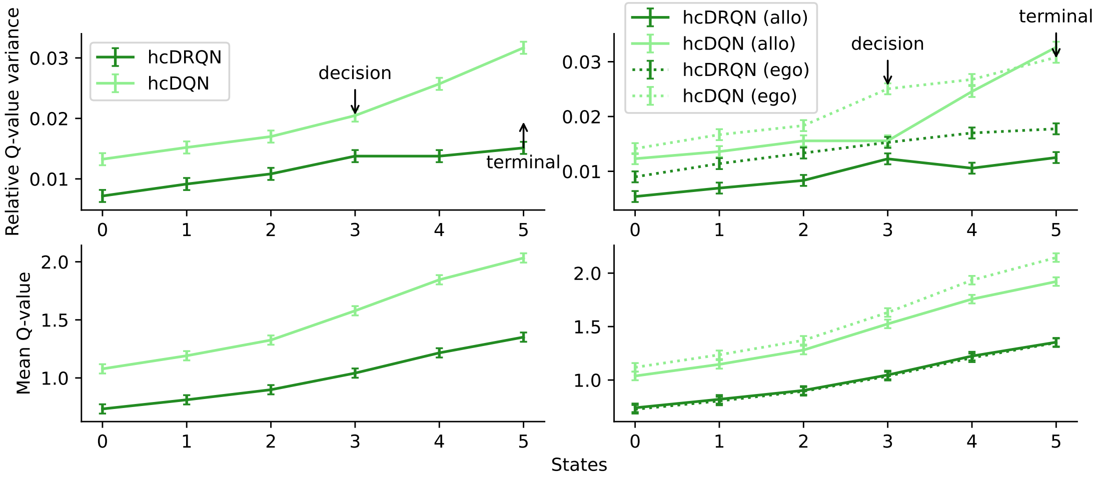


**Supplementary Figure S17. State-dependent action values for full view model.** Top: Relative Q-value variance, given by the variance over Q-values for each state divided by the mean Q-value for each state, shows that hcDQN has higher overall relative variance compare to hcDRQN. Bottom: Average Q-values. Data are presented as mean values ± SEM ($n = 5$). Source data are provided as a Source Data file.

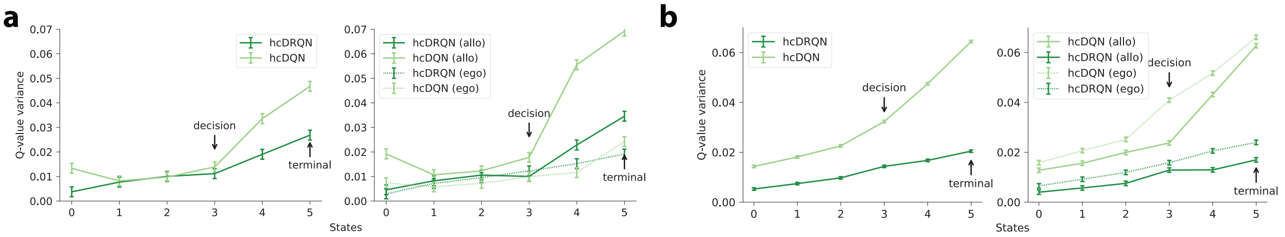


**Supplementary Figure S18. Q-value variance for partial and full view models.** **a**, Partial view: the Q-value variance given by the variance over Q-values for each state. **b**, Full view's Q-value variance. Data are presented as mean values ± SEM ($n = 5$). Source data are provided as a Source Data file.

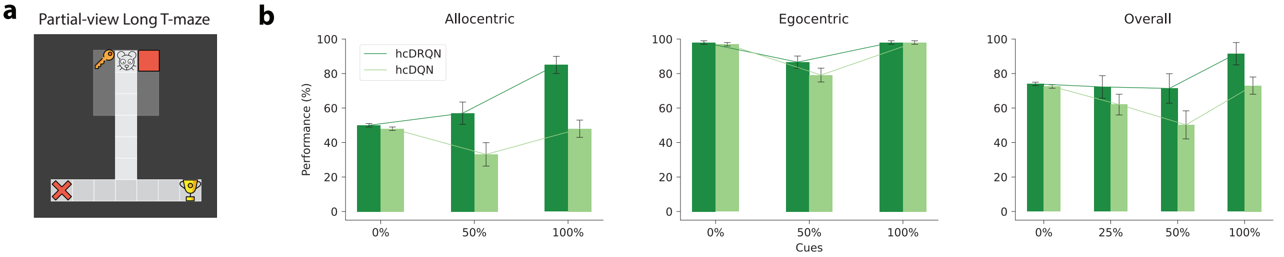


**Supplementary Figure S19. Cue removal with long T-maze and partial view.** **a**, Partial view in a maze with a long corridor. **b**, Allocentric and egocentric task performance. Data are presented as mean values ± SEM ($n = 5$). Source data are provided as a Source Data file. Icons used in panel a are released as open-source by OpenMoji.
