# Supplementary material for: Hippocampus supports multi-task reinforcement learning under partial observability
Source: Nat Commun. 2025 Oct 30;16:9619. doi: 10.1038/s41467-025-64591-9 (PMC12575782; doi:10.1038/s41467-025-64591-9)
Supplement: Supplementary file 2 — Description of Additional Supplementary Files [file 41467_2025_64591_MOESM2_ESM.pdf]

Description of Additional Supplementary Files:

Supplementary Movie 1: Example of sequence of steps taken by our deep RL agent (north starting location).

Supplementary Movie 2: Example of sequence of steps taken by our deep RL agent (south starting location).
