# Supplementary figures and images for: Hippocampus supports multi-task reinforcement learning under partial observability
Source: Nat Commun. 2025 Oct 30;16:9619. doi: 10.1038/s41467-025-64591-9 (PMC12575782; doi:10.1038/s41467-025-64591-9)

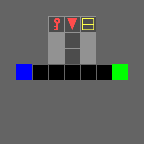

Supplement: Supplementary file 3 — Supplementary Movie 1 [file 41467_2025_64591_MOESM3_ESM.png]

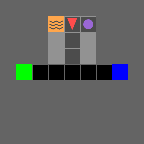

Supplement: Supplementary file 4 — Supplementary Movie 2 [file 41467_2025_64591_MOESM4_ESM.png]
